# In vivo fluorescence imaging: success in preclinical imaging paves the way for clinical applications

**DOI:** 10.1186/s12951-022-01648-7

**Published:** 2022-10-15

**Authors:** Ahmed Refaat, May Lin Yap, Geoffrey Pietersz, Aidan Patrick Garing Walsh, Johannes Zeller, Blanca del Rosal, Xiaowei Wang, Karlheinz Peter

**Affiliations:** 1 Atherothrombosis and Vascular Biology Laboratory, Baker Heart and Diabetes Institute, Melbourne, VIC Australia; 2 Molecular Imaging and Theranostics Laboratory, Baker Heart and Diabetes Institute, Melbourne, VIC Australia; 3 Department of Engineering Technologies, Swinburne University of Technology, Melbourne, VIC Australia; 4 Pharmaceutics Department, Faculty of Pharmacy, Alexandria University, Alexandria, Egypt; 5 Burnet Institute, Melbourne, VIC Australia; 6 Department of Cardiometabolic Health, University of Melbourne, Melbourne, VIC Australia; 7 Department of Medicine, Monash University, Melbourne, VIC Australia; 8 Department of Plastic and Hand Surgery, University of Freiburg Medical Center, Freiburg, Germany; 9 School of Science, RMIT University, Melbourne, VIC Australia; 10 Baker Department of Cardiovascular Research, Translation and Implementation, La Trobe University, Melbourne, VIC Australia

**Keywords:** Fluorescence imaging, ICG, Near-infrared, Antibody conjugates, Targeted imaging

## Abstract

Advances in diagnostic imaging have provided unprecedented opportunities to detect diseases at early stages and with high reliability. Diagnostic imaging is also crucial to monitoring the progress or remission of disease and thus is often the central basis of therapeutic decision-making. Currently, several diagnostic imaging modalities (computed tomography, magnetic resonance imaging, and positron emission tomography, among others) are routinely used in clinics and present their own advantages and limitations. In vivo near-infrared (NIR) fluorescence imaging has recently emerged as an attractive imaging modality combining low cost, high sensitivity, and relative safety. As a preclinical tool, it can be used to investigate disease mechanisms and for testing novel diagnostics and therapeutics prior to their clinical use. However, the limited depth of tissue penetration is a major challenge to efficient clinical use. Therefore, the current clinical use of fluorescence imaging is limited to a few applications such as image-guided surgery on tumors and retinal angiography, using FDA-approved dyes. Progress in fluorophore development and NIR imaging technologies holds promise to extend their clinical application to oncology, cardiovascular diseases, plastic surgery, and brain imaging, among others. Nanotechnology is expected to revolutionize diagnostic in vivo fluorescence imaging through targeted delivery of NIR fluorescent probes using antibody conjugation. In this review, we discuss the latest advances in in vivo fluorescence imaging technologies, NIR fluorescent probes, and current and future clinical applications.

**Graphical Abstract:**

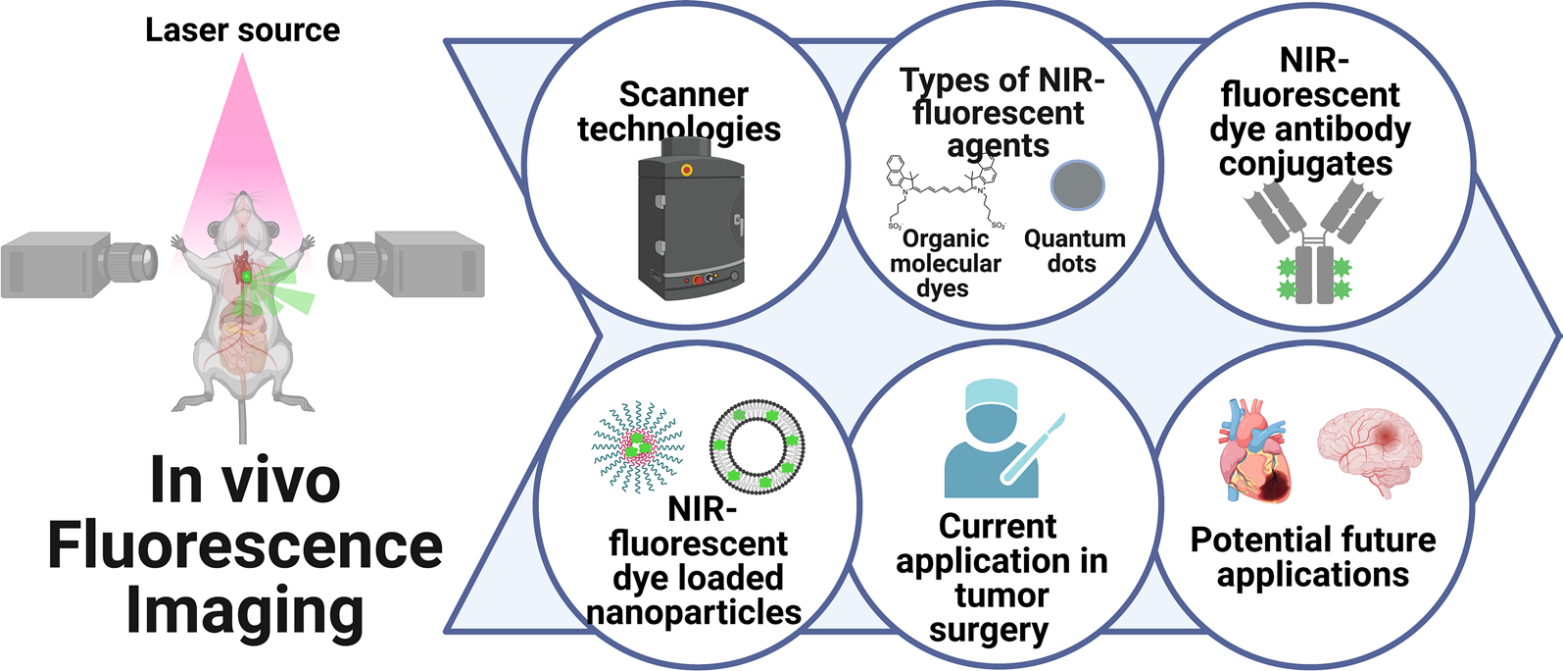

## Introduction—advances in molecular imaging and status of in vivo optical/fluorescence imaging

Progress in the design, synthesis, brightness, and stability of contrast agents, along with technical innovations in imaging technologies, has resulted in rapid progression of molecular imaging for non-invasive visualization, characterization, and quantification of biological processes in humans and other living systems [1–3]. Improvements in sensitivity and spatiotemporal resolution in several molecular imaging modalities have helped to unravel crucial cellular and whole-body processes. Molecular imaging can be used to simultaneously acquire molecular, functional, and anatomical information, making it superior to conventional ex vivo techniques for preclinical research [4].

Currently, clinically applicable imaging modalities include magnetic resonance imaging (MRI), X-ray computed tomography (CT), single-photon emission computed tomography (SPECT), positron emission tomography (PET), ultrasound (US), and optical imaging (OI) [5]. The choice of imaging modality depends primarily on the areas to be imaged and the disease in question, as each technique has its strengths and limitations. For instance, MRI is used to produce excellent contrast for soft-tissue pathologies and is therefore commonly used in studies of the central nervous system [6–8]. However, contrast agents—mainly gadolinium complexes, superparamagnetic iron oxide particles, or Flourine-19—are required in some cases to increase detection sensitivity [9, 10]. The safe use of these contrast agents in patients with impaired renal functions is controversial. A gadolinium-containing contrast agent may pose a risk of severe nephrogenic fibrosis in patients with severe renal failure [11]. CT has a similarly high spatial resolution (0.5–2 mm) and is widely used for studying bone structure. However, its low sensitivity and its reliance on ionizing radiation are major drawbacks. Radionuclide imaging techniques—PET and SPECT—are highly sensitive and can detect biochemical changes and quantify molecular targets. Both require very small amounts (nano- to picograms) of contrast agents, which are therefore unlikely to exert unwanted pharmacological effects. The main limitations of radionuclide imaging techniques are their relatively low spatial resolution (6–10 mm) and the exposure of patients and operators to ionizing radiation. US, while relatively cost-effective and portable, has a limited penetration depth and low resolution. OI has the advantages of relative safety, since it relies on visible or NIR light instead of ionizing radiation, and comparatively lower cost. Some OI techniques (e.g. optical coherence tomography, OCT) are already widely used in the clinic, while others (photoacoustic imaging, Raman spectroscopy, fluorescence imaging) remain mostly preclinical tools. Hybrid imaging systems incorporating two or more imaging modalities that provide complementary information are particularly attractive as they can provide high-quality structural and functional information simultaneously [12, 13].

Fluorescence imaging is a high-sensitivity technique that allows for multiplexed imaging—that is, imaging of multiple different molecules or structures—by using different fluorophores. Compared to other imaging techniques, fluorescence provides real-time imaging, since image reconstruction and post-processing are usually not necessary. However, the limited penetration depth of light into tissues (micron to cm, depending on the wavelength) remains a significant obstacle to its clinical translation. Fluorescence imaging has gained ground as a powerful preclinical technology. Developments in fluorescence imaging probes have resulted in brighter contrast agents which can be detected at extremely low (pico- to femto-molar) concentrations [14]. Conjugating fluorophores, including fluorescent nanoparticles (NPs), to target-specific ligands has enhanced the sensitivity and specificity of fluorescence imaging. Recently, research efforts have focused on developing fluorescent contrast agents with emission bands in the second near-infrared window (NIR-II, 1000–1700 nm) and optical instrumentation for imaging in this spectral range, where tissue transparency is highest [15]. Here we discuss the latest advances in in vivo fluorescence imaging including both imaging technologies and fluorescent probes. We also discuss current and potential future clinical applications and identify the major hurdles for clinical translation of recent research advances.

## In vivo fluorescence imaging

### Historical development of fluorescence imaging and current status

Fluorescence imaging requires an external light source (a lamp, laser, or LED) to optically excite a fluorophore. The emitted fluorescence is then detected using an OI system consisting—in the simplest case—of a lens system, optical filters, and a camera with a charge-coupled device (CCD) or complementary metal oxide (CMOS) sensor. Fluorescence imaging relies heavily on exogenous contrast agents, although many biomolecules present in biological tissues show intrinsic fluorescence (autofluorescence) that can be exploited for diagnostic purposes.

Since the first observation of fluorescence by Fredrick W. Herschel [16] in 1845, the use of fluorescence imaging in biological and medical research has evolved dramatically specially after the development of fluorescence microscopy in early 1900s [17]. The discovery of green fluorescent protein (GFP) in 1962, and its cloning in 1992, represents a great milestone in the journey of fluorescence imaging development [18, 19]. Over the past few decades, technical advances in instrument designs and synthetic fluorescent probes have boosted the use of fluorescence imaging in biomedical research. In vivo fluorescence imaging represents a powerful technique for researchers to study live events using either intravital microscopy [20, 21] or whole animal imaging [22–24]. In preclinical settings, in vivo fluorescence imaging of small animals has been used to quantitatively screen diseases, diagnose and monitor post-treatment evolution. However, clinical application of fluorescence imaging is still limited to a small number of applications, mainly image-guided surgery of tumors. The first reported fluorescence-guided surgery was performed in 1948 using intravenous fluorescein for neurosurgery of intracranial neoplasms [25]. In 1959, indocyanine green (ICG) was approved by FDA for human use as an angiography agent. Since then, ICG has been the preferred angiography agent in some intra-operative applications such as retinal angiography, senile lymph node mapping, and solid tumor resection. This was encouraged by the high safety index, short circulation half-life, and rapid hepatic clearance of ICG [26]. Yet, progress in clinical translation of in vivo fluorescence imaging, as a diagnostic tool, is slow and hampered by many limitations which we will discuss in detail.

### Advantages and limitations

In vivo fluorescence imaging can resolve cellular and subcellular structures using intravital fluorescence microscopy [27, 28] but also enables whole-animal imaging at a sub-mm spatial resolution. The penetration depth depends largely on excitation and imaging wavelengths—whereas visible light will only penetrate a few hundredths of µm, NIR wavelengths allow for imaging at depths of up to a few cm [29]. Fluorescent probes emitting in the NIR-II spectral window provide the highest spatial resolution at large tissue depths due to the reduced scattering of NIR-II light by tissues. Autofluorescence is virtually negligible in the NIR-II, which results in an enhanced image contrast. Compared to other imaging techniques, in vivo fluorescence imaging excels given its minimal invasiveness, real-time and multiplexed imaging capabilities, and relatively low cost. Fluorescence imaging usually requires low concentrations (pico- to femto-molar) of fluorophores to generate high-contrast images, which reduces the cost associated with production of probes and the probability of toxicity to patients.

Fluorescence imaging allows for multiplexed imaging—that is, imaging different molecules or structures simultaneously. Choosing fluorophores with non-overlapping emission bands prevents signal bleed-through between imaging channels as long as adequate optical filters are used [30, 31]. Multiplexed imaging is mostly applied in in vitro techniques such as flow cytometry and immunofluorescence, but the same approach can be employed in vivo. For example, Kobayashi et al. showed in two instances the ability to perform multiplexed NIR-fluorescence imaging in a single imaging session using either dendrimers or monoclonal antibodies (mAb) labeled with fluorophores of different colors [32, 33]. The first study utilized spectrally resolved fluorescence imaging of fluorescently labeled dendrimers to perform 5-color near infrared fluorescence lymphatic imaging with high spatial resolution [32]. The second study used a cocktail of three fluorescently labeled mAb to simultaneously diagnose different tumor subtypes in vivo [33]. The technique provided advantages over radionuclide imaging such as simultaneous differentiation of tumor types, enhanced signal-to-background ratio [S/B], and higher safety.

The major limitation of fluorescence imaging is its poor tissue penetration due to photon absorption and scattering, which attenuate visible light by a factor of approximately 10 per cm of tissue [34, 35]. This must be taken into account during image reconstruction for tomographic imaging and is particularly important in multiplexed imaging since attenuation is wavelength dependent. Employing fluorescent probes with identical emission bands but distinct emission lifetimes can overcome this issue. In vivo, multiplexed imaging in the lifetime domain removes the distortion in the data due to the wavelength-dependent light-tissue interaction, making it possible to quantify the relative concentrations of multiple fluorophores [36, 37].

The development of NIR-emitting fluorophores and imaging strategies has been key to improving the tissue penetration depth of fluorescence imaging [38, 39]. NIR light (650–1700 nm) is less absorbed and scattered by biological tissues than visible light. Few biomolecules have absorption bands in the NIR, minimizing light attenuation and autofluorescence, which results in a greater S/B ratio. This is more pronounced at longer emission wavelengths—the best image contrast is achieved in the NIR-II, also known as short-wave infrared (SWIR), which spans the 1000–1700 nm spectral range. Background autofluorescence decreases at longer wavelengths and is virtually non-existent in the 1500–1700 nm spectral band, known as NIR-IIb [40, 41]. Scattering also decreases with increasing wavelength [42], resulting in better spatial resolution. NIR-II fluorescence enables visualization of small blood vessels that cannot be resolved in NIR-I images including through-skull imaging of the brain vasculature with a sub-10 µm spatial resolution [43, 44].

Fluorescence imaging is sometimes combined with an additional imaging modality—typically MRI or CT—that can provide complementary information and, in the case of fluorescence tomography, aid in image reconstruction (see “Tomographic imaging systems” section). Developing multifunctional contrast agents has thus been a major area of research in the past few years. These are usually a combination of fluorescent and magnetic agents—for example, iron oxide—or radionuclide labels, which are sometimes integrated into a single NP. This is the case of the gadolinium-conjugated quantum dots (QDs) reported by Jin et al. [45].

## Instrumentation for small-animal fluorescence imaging

### Planar imaging systems

Planar imaging systems are simple and can be used for 2D fluorescence imaging in real time. They are typically equipped with one or several light sources for optical excitation of one or several fluorophores. The illumination spot is usually several cm in diameter, allowing for whole-body imaging of mice. A CCD camera collects the emitted fluorescence and generates a 2D image. Usually, optical filters are inserted into the optical path before the camera to prevent any scattered or reflected laser light reaching the detector. Optical filters also facilitate the selection of fluorophore-specific emission bands [14, 15]. The main drawback of planar imaging systems is that the depth from which the fluorescence signals originate cannot be determined. However, they provide a quick and straightforward way to estimate the region and source of a specific fluorophore. A popular example of a commercial planar imaging scanner is the In Vivo Imaging System (IVIS)^®^ Lumina series developed by PerkinElmer. Several systems designed for small-animal fluorescence imaging in the NIR-I and NIR-II are already on the market.

#### Planar NIR-I fluorescence imaging systems

The IVIS^®^ Lumina (PerkinElmer) was the first commercial preclinical 2D fluorescence imaging system. The current version of this system (IVIS Lumina III) uses a 150 W halogen lamp for illumination and is equipped with 19 bandpass filters (20 nm bandpass, center wavelength from 420 to 780 nm) to select the excitation wavelength. Seven fluorescence filters (40 nm bandpass, center wavelength between 520 and 845 nm) facilitate the selection of emission wavelength. The signal is detected by a Peltier-cooled 1024 × 1024 pixel CCD camera with a 13 µm pixel size. The maximum spatial resolution ranges from 50 µm—standard imaging mode—to 35 µm with an optional zoom lens add-in. The height of the imaging stage can be adjusted to determine the size of the field of view from 5 × 5 cm to 12 × 12 cm, which enables imaging of up to 3 mice simultaneously. Using the zoom lens reduces the field of view to 2.6 × 2.6 cm. Other PerkinElmer instruments (IVIS Lumina S5 and X5) use larger CCD arrays (2048 × 2048 pixels, 13.5 µm pixel size) to maintain a similar spatial resolution across a larger field of view—up to 20 × 20 cm—for simultaneous imaging of up to 5 mice.

Other companies have developed similar instruments for in vivo visible and NIR-I fluorescence imaging. These include the Photon Imager (Biospace Lab), UVP iBox Studio (Analytik Jena), Pearl^®^ Trilogy (LI-COR Biosciences), NightOWL II LB 983 (Berthold Technologies), Lago, Ami HT, and Kino (Spectral Instruments Imaging), and Visque InVivo ART and InVivo Smart-LF (Vieworks). All systems are based on the same operating principle, but the excitation source, optical filters, and detector type and size vary depending on manufacturer and model. Except for the IVIS and NightOWL, all systems use a set of LEDs emitting at different wavelengths for optical excitation. Typically, at least 4 LEDs (blue, green, red, and white) are used, but some high-end systems (Spectral Instruments Imaging’s Lago) are equipped with up to 14 LEDs operating at wavelengths between 360 and 805 nm. High-end systems also use larger detector arrays (2048 × 2048 pixels instead of 1024 × 1024). This provides a field of view which is large enough—27 × 27 cm in the case of the Visque InVivo ART and 25 × 25 cm in the Lago—to image 10 mice simultaneously.

These systems typically include a warming platform to maintain the temperature of the animals during the experiment and an inlet port for isoflurane anesthesia. Planar in vivo imaging systems have mostly been used in preclinical oncology to monitor cancer progression and metastasis [46, 47] and in biodistribution studies to investigate the localization of fluorescently conjugated molecular targets and inorganic fluorescent NPs in vivo and ex vivo [48].

#### Planar NIR-II fluorescence imaging systems

As discussed earlier, fluorescence imaging in the NIR-II spectral range (1000–1700 nm) offers the best possible image resolution at large tissue depths (up to ∼1 cm). Technological challenges associated with NIR-II imaging—especially the high cost and relatively low quality of NIR-II-sensitive cameras—have prevented its widespread implementation. NIR-II imaging requires using InGaAs cameras, which need deep cooling to obtain an acceptable S/B. InGaAs detectors also have a lower pixel resolution—640 × 512 pixels for higher-end cameras—than the silicon detectors used for visible/NIR-I imaging.

Commercial NIR-II imaging systems for preclinical research have entered the market only recently, so research groups have relied on their own custom-made systems for in vivo NIR-II fluorescence imaging. The IR-VIVO, developed by Photon etc., is one of the few commercial preclinical imaging systems designed for NIR-II imaging. It is equipped with up to 4 NIR lasers (operating at wavelengths between 670 and 1064 nm) for optical excitation and a Peltier-cooled 640 × 512 pixel InGaAs camera to collect the emitted fluorescence. There are two versions of the IR-VIVO—a multispectral imager equipped with up to 6 filters mounted on a filter wheel and a hyperspectral imager where the fluorescence filters are replaced by a hypercube with a 4 nm resolution. With the hyperspectral imager, a fluorescence spectrum is acquired for each pixel in the image. This permits, for example, identifying small differences in the spectral profiles of the autofluorescence of different tissues. The PhotonIMAGER SWIR, commercialized by Biospace Labs, and the NIR-II Kaer Imaging System (Kaer Labs) offer NIR-II preclinical imaging systems similar to the multispectral version of IR-VIVO.

### Tomographic imaging systems

Tomographic imaging systems allow for 3D in vivo fluorescence and bioluminescence imaging. From a hardware standpoint, tomographic and planar imaging systems have many identical elements (excitation source, excitation and fluorescence filters, and CCD camera) with tomographic systems also incorporating a structured illumination source to image the surface topography of the sample. Tomographic systems rely on a computational algorithm to generate a 3D image from 2D fluorescence images and sample topography. This algorithm models light propagation through tissues—taking into account both absorption and scattering—and needs to consider the properties [excitation cross-section and quantum yield (QY)] of the fluorophore as well. Calibrating each fluorophore (i.e. generating a correlation between fluorophore concentration and fluorescence intensity) is essential in order to obtain quantitative data from tomographic images. Tomographic imaging is a very complex computational problem, which explains the limited availability of commercial tomographic fluorescence imaging systems compared to their planar counterparts.

PerkinElmer has commercialized two series of fluorescence tomography imaging systems—the IVIS^®^ Spectrum and the FMT. The Spectrum series is similar to the IVIS Lumina S5 described in the previous section, using a high-resolution (2048 × 2048 pixel) CCD camera for fluorescence imaging and enabling simultaneous imaging of up to 5 mice. A similar halogen lamp is used for optical excitation and optical filters are used to collect images in different excitation/emission bands between 415 and 850 nm. This allows for multiplexed fluorescence imaging and also simultaneous fluorescence and bioluminescence imaging, as demonstrated by several groups studying oncological [49, 50], cardiovascular [51–53], and rheumatic diseases [54, 55]. FMT systems focus exclusively on NIR-I tomography, with up to 4 lasers (635, 670, 745, and 790 nm) for optical excitation. In both systems, the excitation beam is raster scanned in transmission geometry and a CCD camera placed above the animal stage collects the emitted fluorescence. Although the IVIS Spectrum also allows for imaging in reflection geometry, transillumination can improve quantification and contrast [56].

### Multimodal whole body imaging systems

Combining fluorescence tomography with imaging modalities that provide structural information—especially CT—can aid in image reconstruction. Besides imaging surface topography accurately, CT can distinguish between tissues with different light scattering coefficients, helping to create a scattering map for more accurate 3D fluorescence imaging.

Several commercial units offer combined micro-CT (µCT) and fluorescence tomography, including the IVIS^®^ Spectrum CT (PerkinElmer), U-OI (Optical Imaging Units, manufactured by Milabs), AMI HTX and Lago X (Spectral Instruments Imaging), and InSyTe FLECT-CT (Trifoil Imaging). The working principle of all units is similar, although different manufacturers use different hardware (excitation sources, filters, and detectors) and reconstruction algorithms. Excitation sources can be halogen lamps (IVIS Spectrum CT, U-OI) or LED arrays (Lago X, AMI HTX) for imaging across the visible and NIR-I spectral ranges. The InSyTe FLECT-CT is exclusively focused on NIR-I fluorescence tomography and is equipped with 4 lasers (wavelengths between 642 and 780 nm) for optical excitation.

Instrument throughput also varies greatly depending on the imaging strategy. Units where the fluorescence is collected directly from above the sample stage can have large fields of view that facilitate imaging of up to 5 (IVIS Spectrum CT) or 10 (LagoX) mice simultaneously. The InSyTe FLECT-CT uses a 360-degree imaging approach, where a rotating photodiode array collects all emitted fluorescence—both reflected and transmitted. This can improve the accuracy of image reconstruction but reduces the instrument throughput as only one mouse can be imaged at a time.

**Fig. 1:**
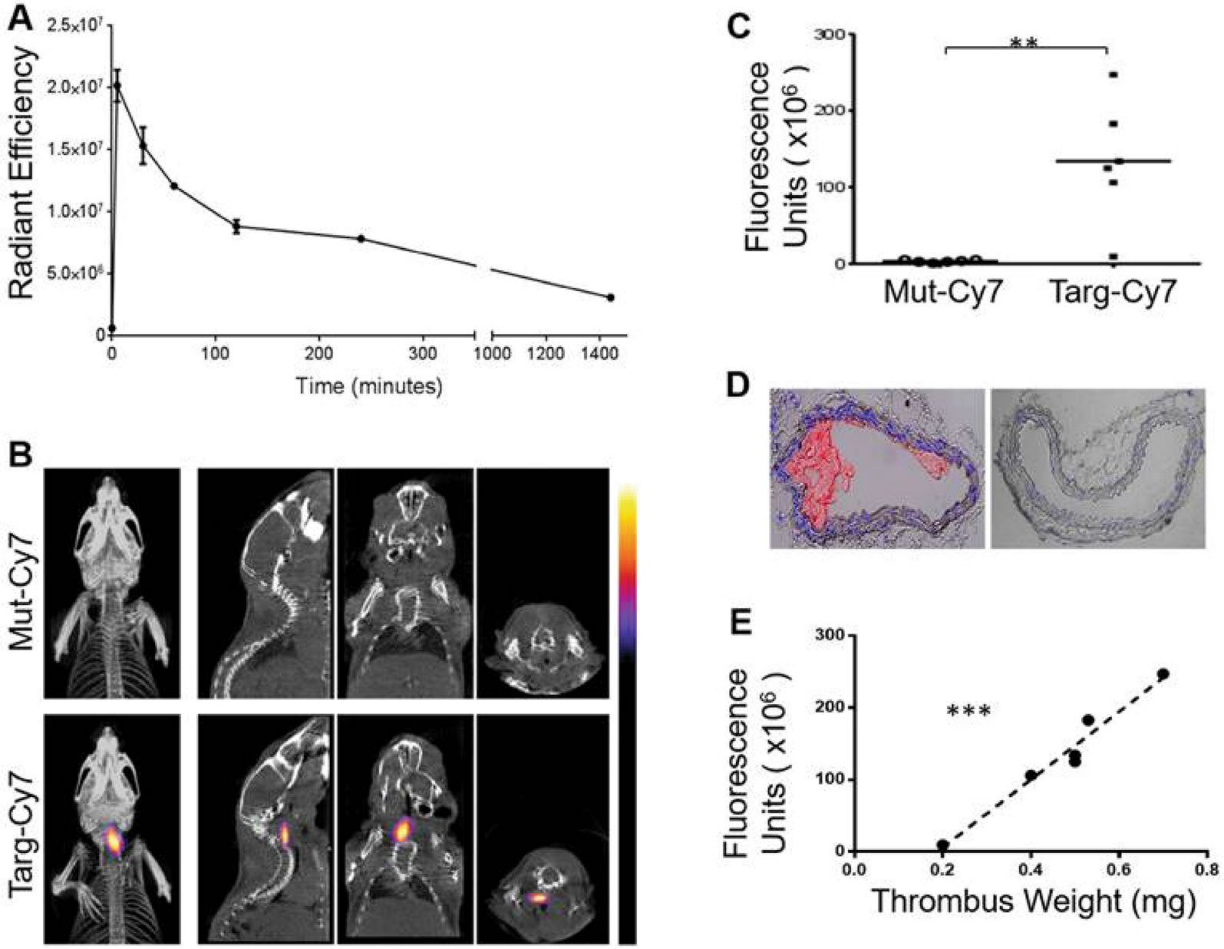
InSyTe FLECT/CT imaging of mice with left carotid ferric chloride–induced thrombosis using a targeted NIR fluorescent fluorophore. **A** NIR fluorescence signal of Targ-Cy7 in collected blood samples as determined by IVIS^®^ Lumina to determine in vivo circulatory half-life before imaging using FLECT-CT. **B** FLECT-CT scans of mice with left carotid thrombosis showing selective binding of targeting fluoroprobe (Targ-Cy7; bottom panel), compared to mutated control (Mut-Cy7; top panel). **C** NIR fluorescence units of Targ-Cy7 and Mut-Cy7. **D** Representative images of ferric chloride–injured carotid artery (left) and contralateral non-injured carotid artery (right), where nuclear stain (DAPI) is blue and platelet-specific (CD41-allophycocyanin) is red. **E** Further analysis of detected signal in each mouse shows a strongly significant correlation to the weight of its ex vivo thrombus. Adapted with permission from [57]. Copyright 2017 Ivyspring

**Fig. 2:**
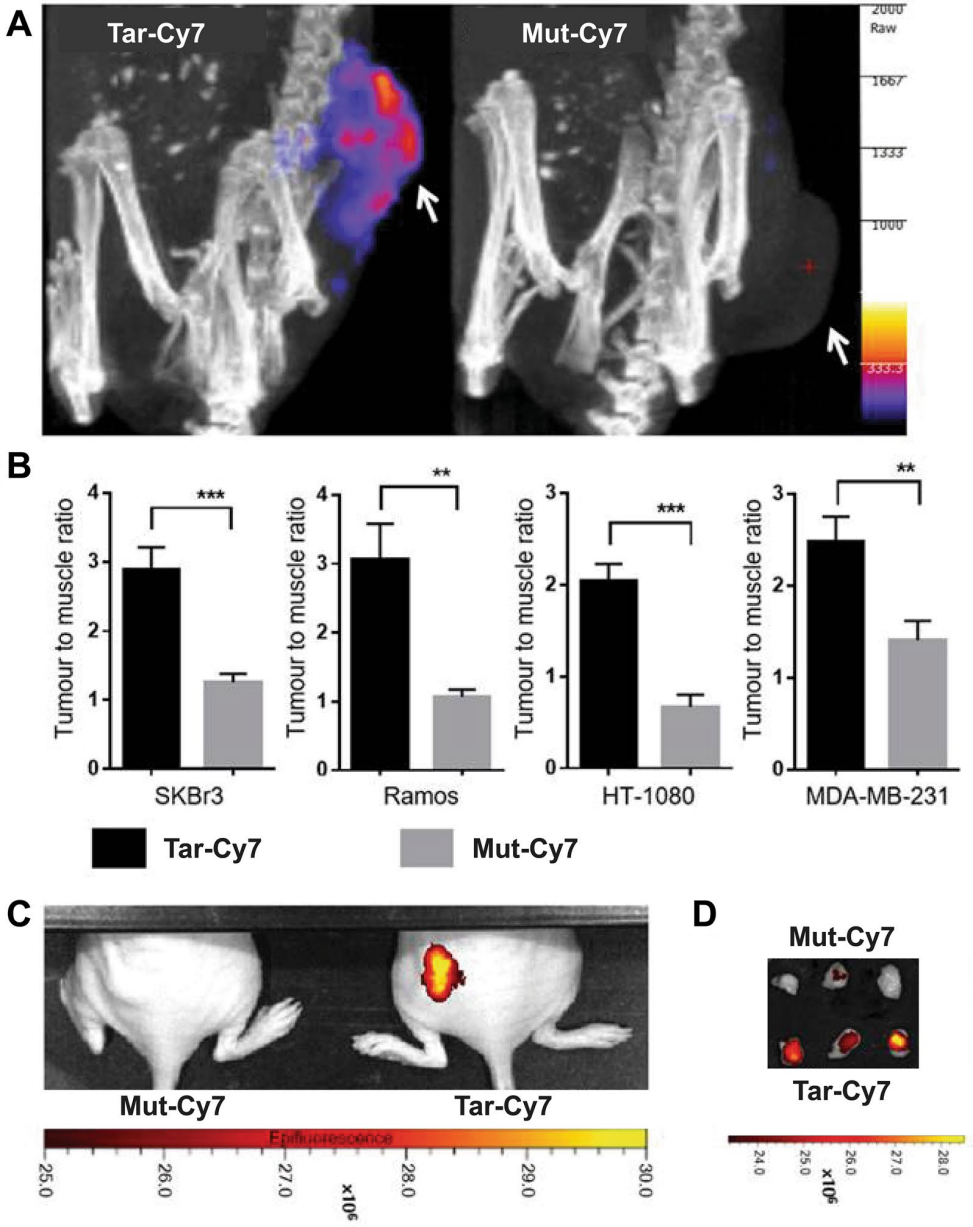
InSyTe FLECT/CT imaging for tumor localization in mice using Targ-Cy7. **A** FLECT imaging of SKBr3 tumor-bearing BALB/c nude mice post injection of Targ-Cy7 or mut-Cy7. **B** Mean fluorescence intensity presented as tumor-to-muscle signal ratio for SKBr3 xenografts, Ramos xenografts, HT-1080 xenografts, and MDA-MB-231 xenografts following injection of Targ-Cy7 or mut-Cy7. **C** 2D IVIS^®^ Lumina scans 20 h following injection of Targ-Cy7 or mut-Cy7. **D** Ramos tumor sections of mice injected with Targ-Cy7 or mut-Cy7 excised and imaged with IVIS^®^. Adapted with permission from [58]. Copyright 2017 Ivyspring

**Fig. 3:**
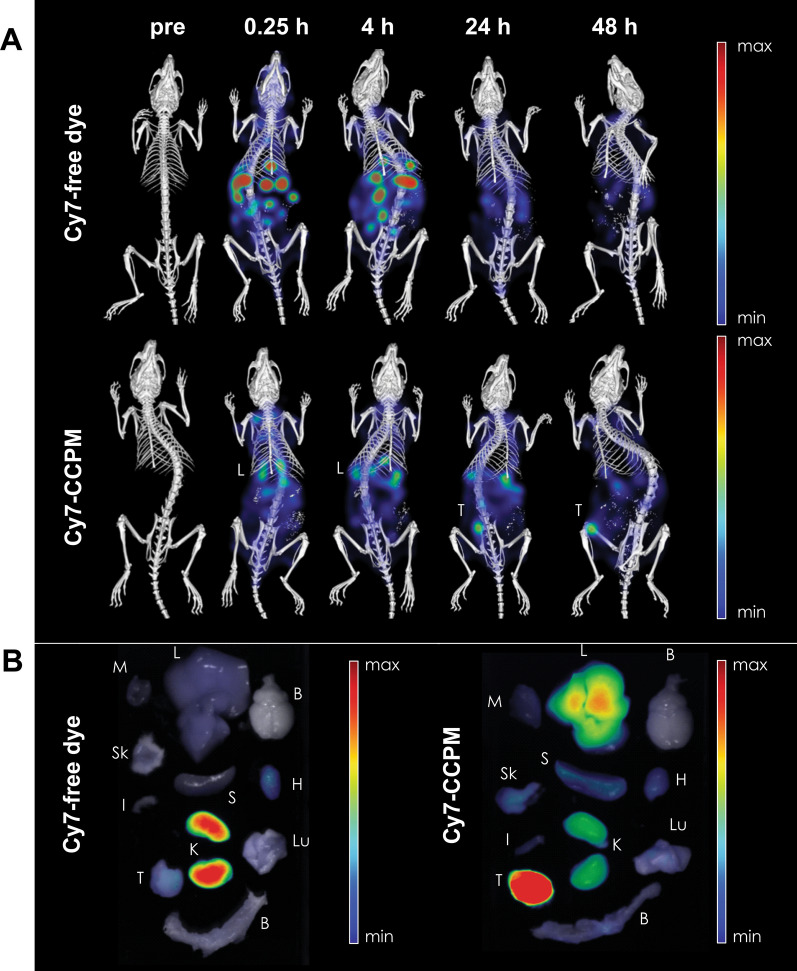
Targeting efficiency of Cy7-labelled CCPM to 4T1 tumors in mice. **A** 3D FLT-μCT imaging of 4T1 tumor-bearing mice following administration of Cy7 as a free dye in comparison to Cy7-CCPM. **B** Ex vivo 2D fluorescence imaging of different organs at 48 h post *i.v.* administration (M = muscle, L = liver, B = brain, Sk = skin, S = spleen, H = heart, I = intestine, K = kidneys, Lu = lung, T = tumor, B = bone marrow). Adapted with permission from [59]. Copyright 2020 Elsevier

The InSyTe FLECT-CT (Trifoil Imaging) has been used in cardiovascular research to diagnose thrombosis in the carotid arteries and lungs [57], as shown in Fig. 1, using cyanine derivative Cy7 dye conjugated with single-chain variable fragment antibody that targets activated platelets. The research group further demonstrated the presence of activated platelets in tumor xenografts as imaged by FLECT-CT (Fig. 2) and provided confirmation via IVIS, PET and US [58]. Another study, by Biancacci et al. [59], used the Milabs FLT-µCT in whole-body imaging to analyze the biodistribution of Cy7-labelled core-crosslinked polymeric micelles (Cy7-CCPM) in mice bearing 4T1 tumors (Fig. 3). The results showed long circulation of the Cy7-CCPM and preferential accumulation in tumors over time, while the free dye (Cy7) was rapidly cleared by the liver and kidneys with low accumulation in the tumor.

Commercially available imaging systems do not always fulfil the growing needs of scientists, although they still represent the majority of fluorescence imaging instruments used in preclinical research. Custom systems developed by research groups incorporate additional features tailored to specific applications, for example time gating for autofluorescence-free imaging in the lifetime domain. Until recently, there were no commercial instruments for in vivo fluorescence imaging in the NIR-II, so a large majority of the research on that topic has been carried out with custom systems.

In the simplest case, custom systems—same as commercial instruments—are equipped with a light source (laser or LED), a set of excitation/emission filters, a focusing lens, and a fluorescence imaging camera. NIR-II imaging systems typically use diode lasers operating at around 800 nm for illumination, since this wavelength shows a good penetration depth into tissues and can excite a variety of organic and inorganic NIR-II fluorophores. High-power (∼20 W) 808 nm lasers are relatively low cost and readily available from a variety of manufacturers, enabling illumination of large surface areas with constant power densities. High-power LEDs (for example, Thorlabs Solis 850 nm LED) are an even more affordable illumination alternative in that wavelength range. NIR-II image acquisition requires cameras equipped with an InGaAs sensors, which require active cooling to reduce the background noise. InGaAs cameras are quite costly (∼$20–$100 k), with the price depending on the sensor size (typically 320 × 256 or 640 × 512 pixels), the readout rate and the cooling capacity. Fan-cooled cameras operating slightly below room temperature are significantly cheaper than Peltier-cooled cameras operating at temperatures close to −80 °C, but will generate lower-quality images with poorer S/B ratios. NIR-II imaging systems typically include notch filters to remove any laser background from the images and longpass filters in the 1000–1600 nm range to select the spectral imaging window. These are sometimes mounted on automated filter wheels to achieve multispectral imaging, with high-speed filter wheels (e.g. Thorlabs FW103H) allowing for filter switching in less than 100 ms. Hyperspectral filter cubes in the NIR-II have become recently available, although they are not commonplace in custom NIR-II imaging systems at this point. Fixed focal length lenses are typically used to form the images in the camera sensors, with relay lens systems sometimes being included when additional optical elements need to be included.

Other optical and electronic elements may be included in the system depending on the specific application. For example, time-gated imaging or lifetime multiplexing requires a pulsed laser source combined with an optical chopper or an electronic delay system to perform time-resolved acquisition. Beyond the optics instrumentation, all custom fluorescence imaging systems are typically equipped with a tubing system for anesthesia and a heating pad to maintain the body temperature of the animal being imaged.

## NIR fluorophores for in vivo imaging

### Organic small-molecule dyes

#### NIR-I fluorescent molecular dyes

**Fig. 4:**
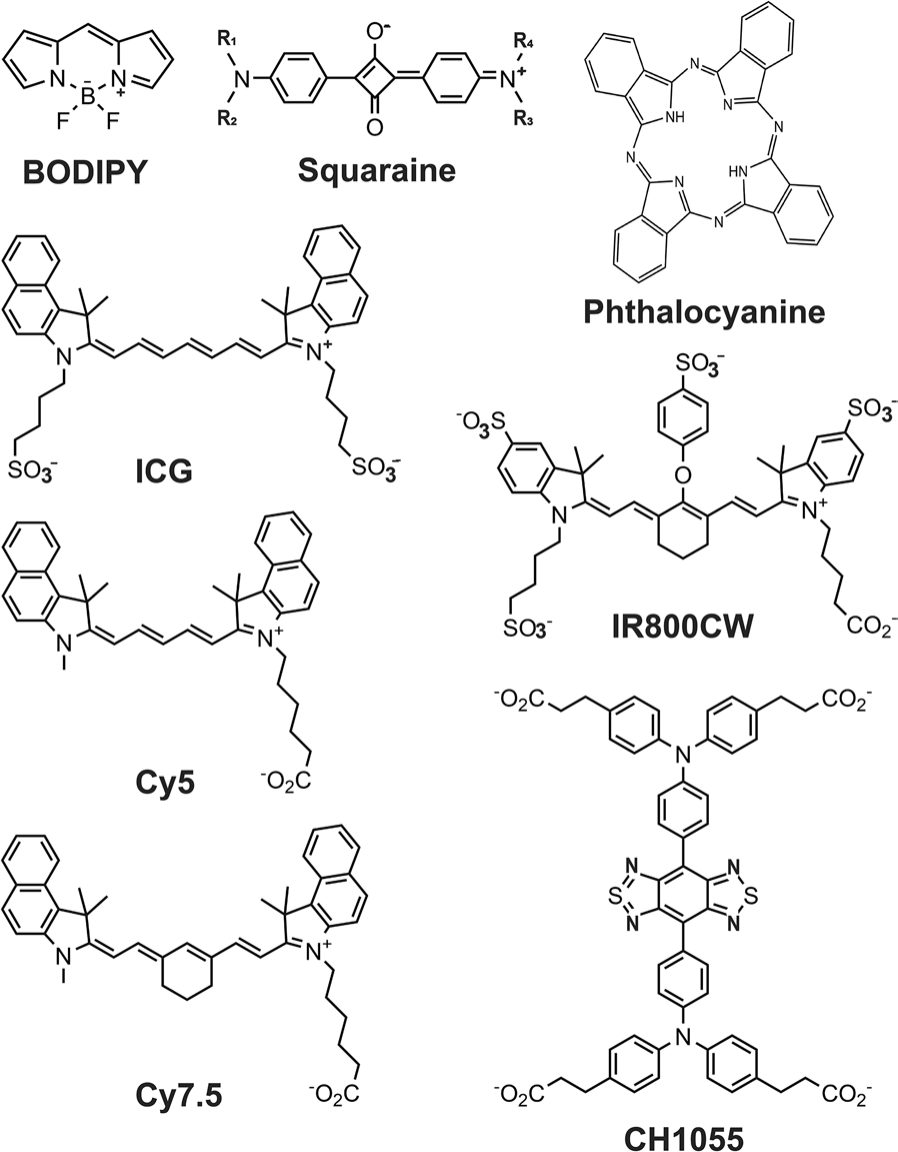
Chemical structures of most commonly used NIR-fluorescent small organic dyes. Heptamethine cyanine dyes are most widely used in preclinical studies. Presence of sulfonate groups and carboxylic acid groups increases solubility and in addition carboxyl group act as activation points for chemical conjugation to ligands

Most NIR-I fluorescent dyes belong to one of the following 4 organic chemical classes: phthalocyanines, squaraines, boron-dipyrromethene (BODIPYs) and cyanines [60–62] (Fig. 4). Of these classes, cyanine derivatives (Cy-7, Cy-7.5) and indocyanine green (ICG) have been most widely used in preclinical imaging, either alone, targeted, or in conjunction with another imaging modality [63]. Until recently, ICG and methylene blue (MB) were the only NIR dyes with FDA approval for clinical use. ICG and MB have excitation/emission bands at 808/830 nm and 664/686 nm, respectively. Both are widely used in fluorescence-guided surgery [64–68] and ICG is also routinely used for vascular imaging of the choroid [69–71]. In late 2021, the FDA approved the use of pafolacianine (Cytalux™), with excitation/emission bands at 776/796 nm, for NIR fluorescence-guided surgery on ovarian cancer. This contrast agent is a folic acid analogue conjugated to a cyanine dye to target the over-expressed folic acid receptors on malignant tumor cells [72].

#### NIR-II fluorescent molecular dyes

Recent research efforts have focused on developing NIR-II contrast agents to take advantage of the lower light–tissue interaction in this spectral window Most novel organic NIR-II fluorophores belong to either the polymethine cyanine (e.g. IR-1040, IR-1048, IR-1051, and IR-1061) or donor–acceptor–donor (D–A–D) architecture (e.g. CH1055) [62]. Chemical structure modification, conjugation, or encapsulation in liposomes or polymeric shells are required to overcome the inherent low QY, poor aqueous solubility, slow clearance, and poor stability in biological fluids of these dyes [15, 62, 73–75]. For example, the low QY of the D–A–D dyes can be mitigated through the introduction of shielding groups such as the S-group to reduce the intermolecular interaction, resulting in brighter fluorescence [76].

While no NIR-II dyes have yet been approved for clinical use, FDA-approved ICG could be repurposed for NIR-II imaging, since the tail of its emission band falls within this spectral window [77]. Starosolsk et al. acquired fluorescence images of the femoral blood vessels in mice in both NIR-I and NIR-II after ICG injection and the S/B ratio was twice as high in the NIR-II images [78]. Similarly, ICG NIR-II imaging has been used in angiography, liver and small-intestine imaging, and lymphatic flow monitoring in mice. These observations are particularly useful as ICG is already approved for clinical use. IRDye800CW, a NIR-I dye widely used in clinical trials, was also shown to be useful in vivo as a fluorophore for NIR-II imaging. Interestingly, both ICG and IRDye800CW showed comparable signals to some of the NIR-II fluorophores such as IR-E1050 [79].

### NIR fluorophore conjugates for improved pharmacokinetics and target-specific imaging

#### NIR fluorophore conjugates for NIR-I fluorescence imaging

FDA-approved dyes (ICG and MB) are useful in angiography and to delineate tumor margins and lymph nodes during surgical excision. However, they are not preferentially targeted to tumors [80]. Achieving tissue selectivity requires developing NIR fluorophores with structure- or function-defined targeting mechanisms [81]. As described in the previous section, targeted NIR dyes have just reached the clinic with the approval of pafolacianine for image-guided surgery [72]. Molecular targeting using antibody technology in particular is well established in both preclinical and clinical settings, with many antibodies already approved by the FDA for cancer immunotherapy such as the anti-epidermal growth factor receptor (anti-EGFR) antibody [82, 83]. In this context, antibodies represent well-established targeting ligands toward enhanced in vivo fluorescence imaging.

**Fig. 5:**
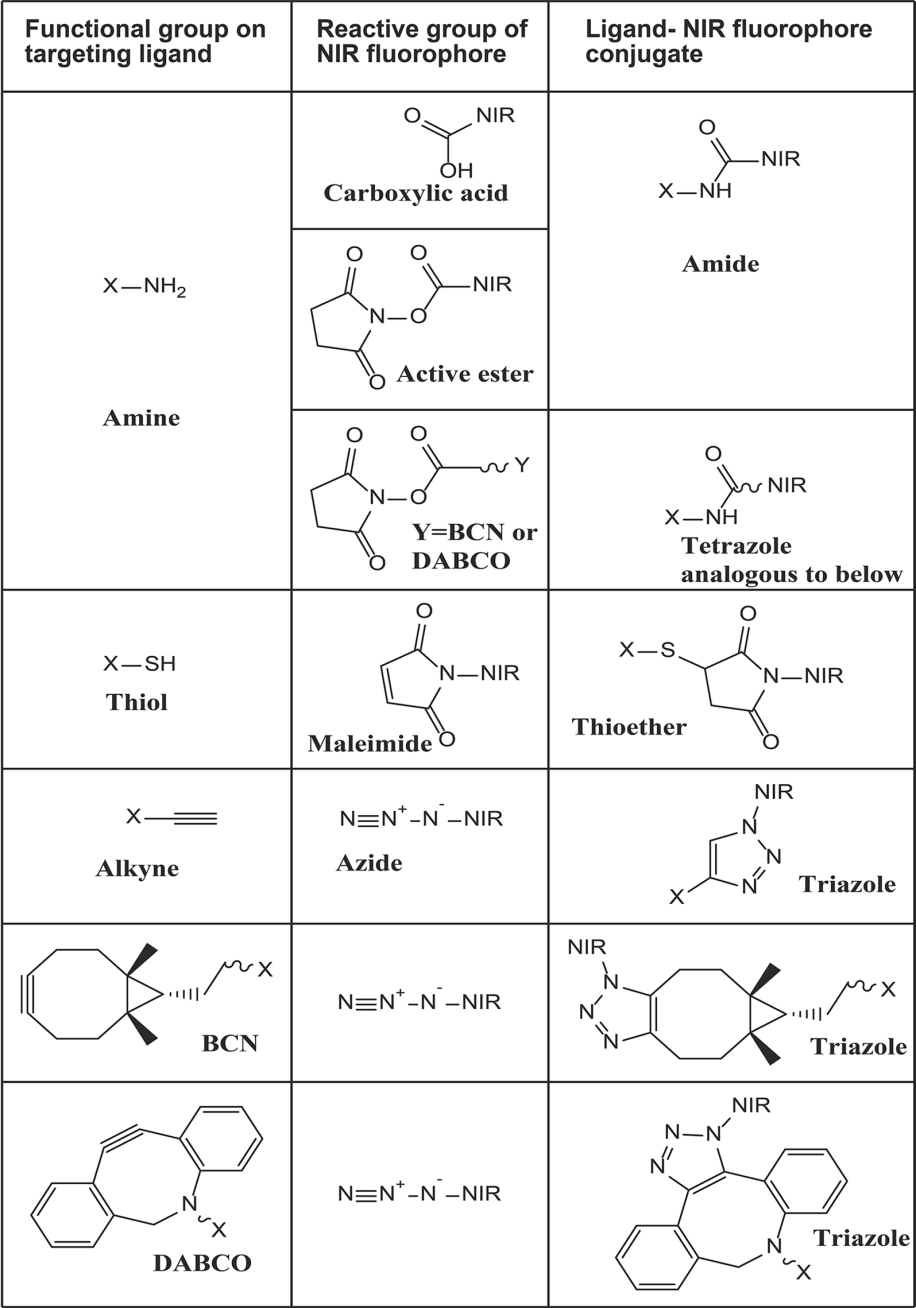
Conjugation strategies for linkage of NIR fluorophores to targeting ligands. X denotes antibody or ligand, and R represents a functional group

Several conjugation methods can be used to fluorescently label antibodies, as shown in Fig. 5. Importantly, conjugation of NIR fluorophores to antibodies should not interfere with their antigen-binding specificity. In antibodies, carboxylic functional groups from aspartic acid and glutamic acid, and amino groups from lysines, are frequently used as attachment points for fluorophores. Conjugation via the carboxylic groups to amine-containing molecules is facilitated by water-soluble condensing agents such as *N*-Ethyl-*N*′-(3-dimethylaminopropyl) carbodiimide hydrochloride (EDC) with or without the addition of N-hydroxysuccinimide. However, this procedure is undesirable due to intra- and inter-molecular crosslinking based on the antibody's lysine amino groups, which may lead to aggregation. Linkage of molecules to antibodies via antibody lysine amino groups using N-hydroxysucciimide ester derivatives, although not site-specific, yields conjugates of heterogenous composition that can be readily controlled or minimized and are thus widely used. Reduction of intrachain disulfides of antibodies to expose sulfhydryl groups that react readily with maleimides is a frequently used strategy due to inherent site specificity without the need to specifically engineer recombinant antibodies or antibody fragments. Similar conjugation can also be carried out without site selectivity by first modifying antibody lysines with a bifunctional thiolation reagent such as succinimidyl 3-(2-pyridyldithio) propionate or iminothiolane. Similarly, alternative crosslinking agents can be used to introduce alkynes or azides for reaction with suitable fluorophores using click chemistry.

Readily available recombinant antibody technology enables the introduction of various functionalities such as sulfhydryl groups, azides, alkynes, and various enzymatic tags, allowing for site-specific linkage of small molecules, and has been extensively reviewed [84]. Figure 5 shows the common conjugation strategies used for NIR fluorophores. Furthermore, small recombinant antibody fragments also facilitate the rapid clearance of tracers, enabling higher target uptake and S/B ratios [57, 58].

While there are many preclinical studies of NIR imaging using targeted conjugates, there are only a few clinical studies. Most of these studies utilize the fluorophore IRDye800CW. The FDA has approved several monoclonal antibodies for immunotherapy, including Bevacizumab against vascular endothelial growth factor-A (anti-VEGF-A), while Cetuximab (anti-EGFR) and Panitumumab (anti-EGFR) have been used with IRDye800CW for image-guided surgery [85–88]. These studies have confirmed the safety and preferential uptake of tracers in tumors enabling resection of malignant tissue. Furthermore, in a clinical study by Rosenthal et al. with a cohort of 12 patients with metastatic head and neck cancer, Cetuximab-IRDye800CW yielded a sensitivity of 97.2% and a specificity of 97.2%, thereby enabling resection of tumor that was missed by the surgeon [88].

#### NIR fluorophore conjugates for NIR-II fluorescence imaging

**Fig. 6:**
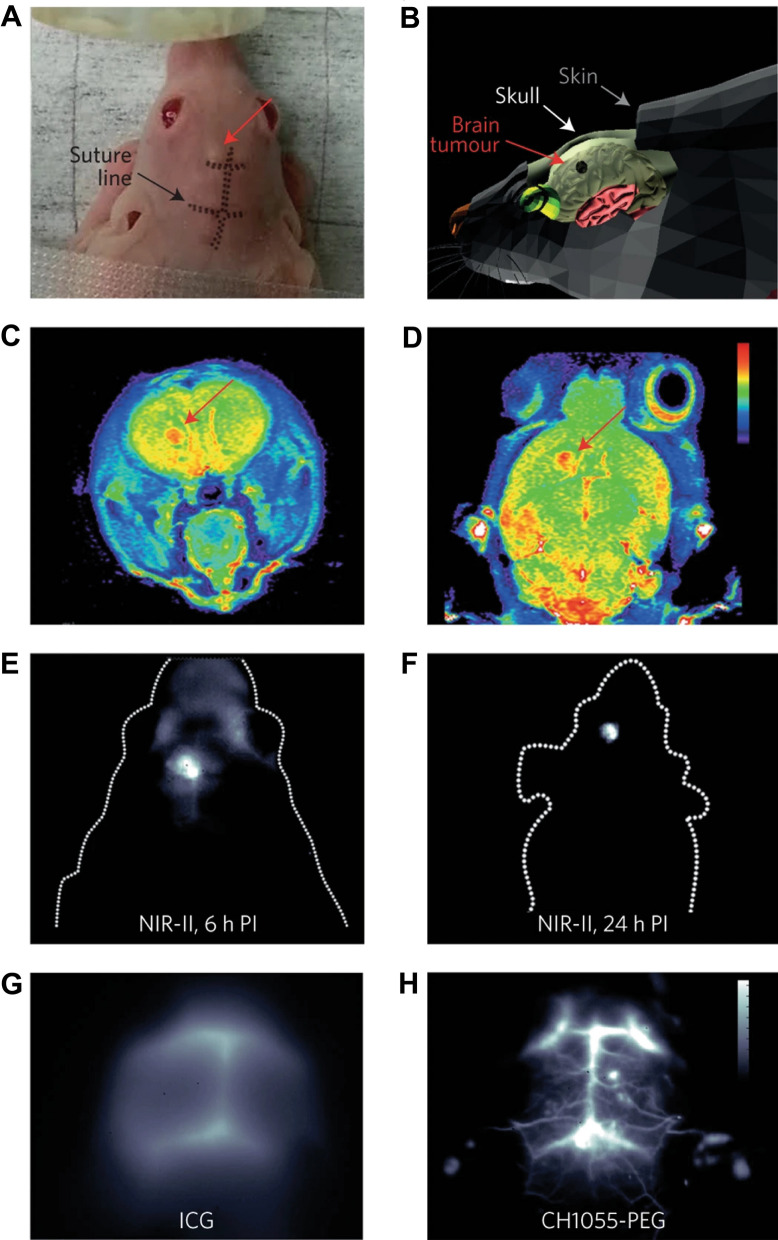
Non-invasive transcranial NIR-II fluorescence imaging of brain tumor using CH1055-PEG. **A** Photograph of a nude mouse before NIR-II imaging. **B** Graphic representation showing the location of brain tumor. T2-colour weighted MRI images of mouse in the sagittal (**C**) and coronal (**D**) planes, showing brain tumors at a depth of ~ 4 mm, immediately before NIR-II fluorescence imaging **E** Transcranial NIR-II fluorescence imaging of brain tumor 6 h post intravenous injection. **F** Whole-body NIR-II fluorescence imaging 24 h post-injection. Brain vasculature imaging through the scalp and skull of C57BL/6 mouse with shaved head using either (**G**) ICG (850–900 nm) or **H** CH1055-PEG (1,300 nm). Adapted with permission from [89]. Copyright (2015) Nature Publishing Group

Conjugating NIR-II fluorescent dyes can also increase their aqueous solubility, clearance rate, and selective accumulation to the tumor. Antaris et al. developed a rapidly excreted, water-soluble, polyethylene glycol (PEG)-conjugated small-molecule dye CH1055 through the introduction of 4 carboxylic groups into the D–A–D structure followed by conjugation to PEG-2000 via EDC/NHS chemistry [89]. The developed PEG-CH1055 showed rapid renal clearance within 24 h and high uptake into brain tumors of mice as demonstrated by the high tumor S/B through intact skulls (Fig. 6). The same group further tested CH1055 conjugation to an anti-EGFR antibody for specific targeting of squamous cell carcinoma tumor xenografts in mice to achieve 5 times higher signals than with NIR-I imaging.

### NIR-fluorophore-encapsulated nanoparticles for enhanced imaging, targeted delivery, and theranostic application

Since most NIR-fluorescent organic dyes are hydrophobic, encapsulating them in amphiphilic phospholipid- or polymer-based nanocarriers enhances compatibility with biological fluids, preventing aggregation and increasing photostability. Jiang et al. used human serum albumin to prepare biocompatible NPs loaded with IR780, a hydrophobic NIR-fluorescent dye, using a protein self-assembly method [90]. The prepared IR780-encapsulated NPs showed higher hydrodynamic stability and photostability in aqueous medium compared to the free dye. Similar observations were reported when IR780 was encapsulated in different types of nanocarriers [91] such as transferrin NPs [92], lipid NPs [93], liposomes [94], and micelles [95]. Wu et al. used an amphiphilic PEG-conjugated phospholipid (DSPE-PEG) to prepare hydrophilic micelles encapsulating the hydrophobic NIR-II fluorescent dye BPST. The newly formed NPs not only enhanced solubility and stability in aqueous medium, but also increased the QY of the incorporated dye for efficient in vivo imaging [96].

Besides enhancing dye stability, loading dyes into NPs drives their passive accumulation in tumors due to the enhanced permeability and retention effect. This localized accumulation significantly increases the S/B ratio and allows for extended monitoring of tumor tissue compared to the free dye [59, 91, 97, 98]. An et al. reported significantly improved tumor accumulation and more efficient NIR fluorescence imaging with ICG-loaded bovine serum albumin self-assembled nanocomplexes (ICG-BSA NC) compared to free ICG [97]. Enhanced hydrolytic stability and increased QY of ICG-BSA NC were also confirmed [97]. Further, conjugating dye-loaded NPs to targeting moieties allows ligand-mediated active targeting. This active-targeting approach has been investigated for NIR fluorescence–guided thrombosis diagnosis [99] and cancer surgery [100]. In addition to stabilization and targeted imaging, NPs can be co-loaded with a dye and a therapeutic agent for theranostic applications. A ubiquitous example is the ICG/doxorubicin co-loaded NPs, which have been widely investigated preclinically for image-guided chemo-photothermal therapy for cancer [101–103].

### Inorganic fluorescent probes

Compared to NIR organic dyes, NIR-fluorescent NPs are more photostable and brighter, and their large surface-to-volume ratio facilitates conjugating targeting ligands. This has motivated significant research efforts on NIR-fluorescent NP development in the past decade. Different types of inorganic NPs—semiconductor QDs, lanthanide-doped nanocrystals, and carbon-based nanomaterials (carbon nanotubes, carbon dots (CDs), and nanodiamonds)—have been used as NIR-I and NIR-II fluorescent contrast agents in preclinical imaging, as reviewed in detail elsewhere [104, 105].

QDs have attracted the most interest for bioimaging due to their high brightness, photostability, and easy tunability of their emission band [106]. QDs have larger absorption cross-sections and QY—particularly in the NIR—than organic dyes [107]. They also present very broad excitation bands, so a single illumination source can optically excite QDs with different emission bands, facilitating multiplexed imaging [108]. As synthesized, QDs are hydrophobic and thus unsuitable for application in living organisms, so a ligand exchange or coating procedure is required to make them stable in aqueous solutions [109]. QDs with NIR-I emission bands typically have a Cd-containing core (usually CdTe or CdS) often coated with a protective shell that enhances the QY [110].

**Fig. 7:**
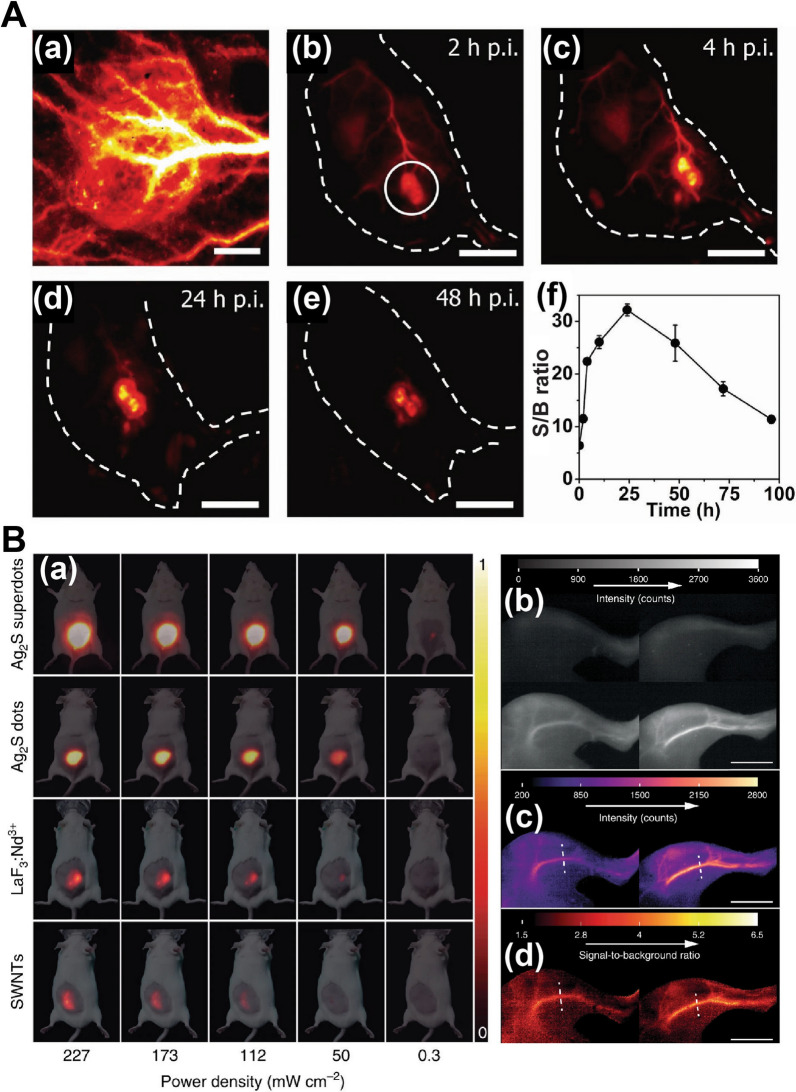
Deep tissue fluorescence imaging in the NIR-II window using QDs. **A** In vivo fluorescence imaging of tumor in the NIR-II window with a S/B > 30. (a) Wide-field fluorescence imaging of a tumor xenograft on a mouse after tail vein injection of PEG-coated core/shell PbS/CdS QDs, showing strong signals for 48 h post-injection (b–e). (f) Time course curve of S/B over 96 h post injection. Adapted with permission from [116]. Copyright (2018) National Academy of Sciences. **B** NIR-II fluorescence imaging of mice using PEG-coated Ag_2_S superdots. (a) NIR-II fluorescence images of four groups of mice subcutaneously injected with colloidal aqueous dispersions containing Ag_2_S superdots, commercial Ag_2_S dots, SWNTs, and LaF_3_:Nd NPs, using different laser power densities. (b) NIR-II fluorescence images of the left hind limbs of two mice immediately before (top) and 15 s after (bottom) of an intravenous injection of commercial Ag_2_S dots (left) or superdots (right). (c) Net intensity images obtained from subtracting the background images (top row in b) from the signal images (bottom row in b). (d) Signal-to-background images obtained by dividing the signal intensity images (bottom row in b) by the background images (top row in b). Adapted with permission from [117]. Copyright (2020) Springer Nature

There are several QD compositions with emission bands in NIR-II, with PbS, InAs, and Ag_2_S the most popular [104]. PbS QDs have relatively high QY and can be synthesized to have emission bands between 900 and 1600 nm, which enables high-resolution autofluorescence-free NIR-IIb imaging [111, 112]. However, Ag_2_S QDs remain the preferred option for bioimaging despite their smaller emission tunability range (900–1220 nm) [113, 114] and lower QY (2% compared to 40% on average for PbS QDs [115] since they lack highly toxic heavy metal ions in their composition. Surface modification of Ag2S nanoparticles with ultrafast photochemistry has been recently shown to improve their QY up to 10%, improving their capacity for high signal-to-background imaging (Fig. 7) [116, 117].

A major limitation on the clinical translation of QDs is their long-term accumulation in vivo and the potential associated toxicity, especially in the case of Pb- and Cd-containing QDs. Core/shell and core/multishell structures, typically with a ZnS outer shell, are often used to prevent the leaching of heavy metal ions [118], although their long-term stability in physiological conditions remains unexplored.

Single-wall carbon nanotubes (SWNTs) are the most researched carbon-based nanomaterial for in vivo fluorescence imaging, since they can be synthesized to show an NIR-IIb emission band [119]. This allows for imaging with micrometric resolution at a tissue depth of several mm. However, their extremely low QY (~ 0.1–1%) and potential long-term accumulation in the body remain major hurdles to their clinical translation [120]. While CDs typically have excitation/emission bands at shorter wavelengths, NIR-II–emitting CDs have been recently developed [121]. Unlike SWNTs, CDs are mostly cleared by the renal pathway in the first few hours after injection, which reduces concern about potential long-term accumulation.

Lanthanide-doped NPs—nanocrystals doped with one or more trivalent lanthanide ions—are extremely photostable and show narrow excitation and emission bands with large Stokes shifts. Their fluorescence mechanism usually relies on an energy transfer process between two different types of ions—an activator, which absorbs the excitation light, and an emitter. Depending on the dopant ions, emission can occur at wavelengths longer (downshifting) or shorter (upconversion) than the excitation wavelength [122]. For fluorescence NIR imaging, lanthanide NPs are usually co-doped with Yb^3+^ ions, which can be excited efficiently with 915 or 980 nm light, and an ion with emission bands in the NIR-I (Tm^3+^) or NIR-II (Er^3+^ and Ho^3+^) [123]. Er^3+^ ions are particularly interesting since their NIR-IIb emission band (1520) allows for imaging small deep-tissue structures—such as brain vasculature—with good (~ tens of µm) spatial resolution [124, 125]. In some cases, only one dopant ion is present—this is usually the case with Nd^3+^-doped NPs, which display two NIR-II emission bands (a more intense one at 1064 nm and a weaker one at 1350 nm) upon excitation at 808 nm [126].

Lanthanide-doped NPs have long fluorescence lifetimes (up to milliseconds) that can be tuned by adjusting the content of dopant ions. This provides multiplexed imaging in the time domain, facilitating quantitative analysis of in vivo images [36]. Since the typical lifetimes of autofluorescence are much shorter (~ ns), lanthanide NPs also allow for real-time in vivo autofluorescence-free imaging using time-gated image acquisition [127].

Besides their superior photostability, inorganic fluorescent NPs have much greater multifunctional potential than molecular probes. For example, some NIR-fluorescent NPs have good photothermal conversion efficiency, enabling their use for simultaneous imaging and localized heating—for example, in photothermal therapy for tumors [128]. Some NIR-fluorescent NPs can also act as in vivo local sensors of physical and chemical parameters, including temperature [129], pH, and presence of ionic or molecular species. Finally, inorganic fluorescent NPs can be designed to offer contrast to additional imaging modalities such as CT or MRI. However, long-term accumulation and potential toxicity remain major limitations for their clinical translation.

## Current and potential clinical applications of in vivo fluorescence imaging

For potential translation and clinical applications, the limitation in penetration depth is a major obstacle. Thus, fluorescence imaging is mainly used in angiography and fluorescence-guided surgery on tumors. Advances in the development of NIR fluorescence probes, guidewires, and scanners may facilitate more efficient deep-tissue imaging, for example, through the skull for brain tumor imaging and for cerebral perfusion assessment in acute stroke patients. Additionally, fluorescence imaging can also be utilized to image areas which are accessible by endoscopy and for intraoperative visualization, as further highlighted below.

### Surgical oncology

Surgical oncology is the main area of application of fluorescence imaging. This development was spurred by the FDA approval of the NIR-fluorescent agent ICG for clinical application [130, 131]. ICG is used in cancer surgery for identifying and defining tumour margins and localizing lymph nodes, to accurately delineate tumor resection margins while sparing surrounding healthy tissues [132–135]. ICG is also used to visualize lymph drainage and sentinel lymph nodes in both plastic surgery, for example, for anal cancer and breast cancer [136, 137], and dermatology [138]. Furthermore, ICG is used in the visualization of blood perfusion of composite tissue in plastic surgery, for example, in reconstructive surgery of the breast with patient-own tissue after mastectomy [139]. Sufficient perfusion of combined skin and fat tissue can be assessed intraoperatively to ensure best operative results.

Recently, the concept of ICG fluorescence retention due to an enhanced permeability in solid tumors was introduced [140]. High-dose, delayed imaging ICG (SWIG) exploits the higher permeability of the tumor vasculature and thus, its poor clearance and retention on site, an effect known as the enhanced permeability and retention effect [141]. Compared to the otherwise fast clearance and short half-life of minutes in the circulation, ICG is trapped on site of the tumor, visualizing the tumor bed. The second window ICG technique has demonstrated its feasibility in a clinical prospective trial localizing glioblastoma in the human brain [142]. Intriguingly, the authors reported a plateau period of up to 30 h for enhanced S/B ratio. However, the increased sensitivity for tumor tissue is accompanied by a decrease in specificity [143]. Likewise, Teng et al*.* reported the ease of localization of brain metastases by SWIG. Both the improved sensitivity in tumor margin detection neoplasm and the transdural localization, i.e., imaging through the outmost of the meninges, the thick membrane of connective tissue called dura mater, facilitating the surgical procedures [144].

Additionally, the development of cancer-targeting fluorescent probes represents a new tool toward more efficient, image-guided, minimally invasive surgery. Meijer et al. detected colorectal and pancreatic liver metastases using a fluorescent probe targeted to carcinoembryonic antigen, a tumor cell marker, during both open and laparoscopic surgery [145]. In another study, Valk et al. imaged colon carcinomas in patients during laparoscopic surgery [146]. The group conjugated cyclic pentapeptide (cRGD), which binds to various integrins expressed on tumor cells, to the NIR fluorophore ZW800-1 and administered it to patients, enabling tumor detection in a minimally invasive laparoscopic experimental design. Hoogstins et al. used intraoperative NIR fluorescence imaging to detect ovarian cancer in 12 patients [147]. The group detected 29% more lesions than by standard clinical practice (inspection and palpation), demonstrating the potential of fluorescence imaging to improve patient outcomes in cancer surgery. Furthermore, endoscopy can help with the placement of fluorescent probes close to the area of suspected disease to facilitate imaging with minimal signal absorption from surrounding tissue. Nagengast et al. employed NIR fluorescence molecular endoscopy (NIR-FME) while performing endoscopic mucosal resection to detect esophageal adenocarcinomas [148]. The research group used a fluorescently labeled (IRDye800CW) antibody against VEGF to improve the sensitivity of lesion detection. Similarly, de Jongh et al. used NIR-FME to detect lesions in 15 patients with Barrett’s esophagus [149].

These advances come hand in hand with the generation of novel and portable fluorescence imaging systems, which are paving the way for further development and progression of in vivo fluorescence imaging in cancer surgery. For breast cancer screening, the FLARE (fluorescence-assisted resection and exploration) system was one of the first intraoperative imaging devices tested, in preclinical trials in 2009, for sentinel lymph node mapping of patients with breast cancer [150]. Later, a portable optical device called the photodynamic eye NIR fluorescence imager was developed for imaging of peritoneal metastases [151]. Another example of a portable device is the Fluobeam^®^ system, developed by Fluoptics in France. It has been tested for mapping of peritoneal carcinomatosis in colorectal cancer patients using ICG as a fluorescent agent and shown to correctly map metastatic areas with a sensitivity of 97% and a test accuracy of 95.6% [152].

### Cardiovascular and cerebrovascular diseases

Fluorescence imaging has recently attracted attention as a potential diagnostic tool for vulnerable, rupture-prone atherosclerotic plaque, relying on its NIR autofluorescence (NIRAF). A major advantage is that its detection is inherent and occurs without the need to administer exogenous fluorophores. Htun et al. demonstrated that NIRAF can be used to characterize high-risk, rupture-prone plaques with intraplaque hemorrhage in both mice and humans [153]. The group identified the potential source of NIRAF to be bilirubin, which is generated by heme degradation at the site of intraplaque hemorrhage in unstable plaques [153]. This discovery might pave the way for development of a reliable, intracoronary fluorescence imaging technique to identify dangerous, rupture-prone plaques in the coronary and carotid arteries that can then be stabilized by either highly effective but potentially expensive medical therapy (e.g. PCSK9-inhibitors) or stenting. Indeed, NIRAF as a marker of plaque instability has been validated in the coronary arteries of patients [154–156].

Another potential application of NIR fluorescence imaging is monitoring cerebral perfusion in stroke patients as an extension of the widely investigated time-resolved near-infrared spectroscopy (trNIRS). Steinkeller et al. used ICG bolus and trNIRS for bedside monitoring of cerebral perfusion in acute stroke patients [157]. Further, Weigl et al. demonstrated the versatility of ICG in the assessment of brain perfusion in a cohort of post-traumatic brain injury patients [158]. Recently, Saito et al. used ICG and trNIRS to examine patients with occlusive cerebrovascular disease [159].

### Other potential applications

Beyond image-guided surgery, NIR fluorescence imaging with ICG as a contrast agent is frequently used to visualize disturbed lymphatic drainage, for example, in secondary lymphedema following surgery [160]. It is also diagnostically applied in lymphangiography, informing the treatment of lymphedema and the reconstruction of lymph vessels. In addition, fluorescence imaging can be used to identify parathyroid glands during thyroid surgery, reducing the chance of incidental parathyroidectomy [161]. This is done by relying on the NIR autofluorescence of the parathyroid glands, although using ICG as a contrast agent improves the S/B ratio [162].

Akram et al. used optical endomicroscopy to image gram-negative bacteria in patients with bronchiectasis and those with suspected pneumonia under mechanical ventilation in ICU [163]. The group used polymyxins (PMX), which bind to gram-negative bacteria, conjugated to the fluorophore 7-nitrobenz-2-oxa-1,3-diazole (NBD) to provide the signal. During bronchoscopy using a fiber optic bronchoscope, NBD-PMX was topically administered before imaging. Gram-negative bacteria were successfully detected in the distal lungs of multiple patients; however, optimization for differentiating between non-pathogenic colonization and infection is required. Further, an opportunity exists to expand imaging to additional pathogens including gram-positive bacteria, resulting in a robust screening modality for patients at risk of pulmonary infection.

## Conclusion and future perspectives

The development of molecular imaging in biomedical research is playing a pivotal role in elucidating the pathogenesis and diagnosis of diseases such as cancer, atherosclerosis, and infectious diseases. In preclinical research, fluorescence imaging has become a highly valued tool for investigating disease mechanisms, as well as testing new diagnostic and therapeutic reagents. These advances have been achieved by exciting developments in two areas: (1) the generation of new and better fluorophores, and the associated introduction of novel conjugation chemistries; and (2) the development of several in vivo preclinical imaging systems which overcome the limitations of autofluorescence, light scattering, and attenuation, as described in this review. The development of multimodal imaging systems incorporating fluorescence imaging is one of the cornerstones for future technologies which will allow for further development of superior systems with high spatial and temporal resolution. In parallel, continuing developments of new algorithms are helping to overcome the limitations of the current imaging systems, especially in regard to the depth of penetration. In preclinical biomedical research, fluorescence imaging has developed into a broadly available and more and more frequently used technology due to its sensitivity, cost-effectiveness, and safety, along with the availability of a vast variety of functionalized fluorophores. Initial uses in the diagnosis and therapy of cancer and in directing surgical approaches serve as indicators of the future potential of fluorescence imaging in clinical medicine. With technological advances, fluorescence imaging is expected to play an increasing role in the clinical management of patients, thereby facilitating imaging-driven personalized medicine.

## Data Availability

Not applicable.

## Abbreviations

NIR: Near infrared
ICG: Indocyanine green
MRI: Magnetic resonance imaging
CT: Computed tomography
SPECT: Single-photon emission computed tomography
PET: Positron emission tomography
US: Ultrasound
OI: Optical imaging
OCT: Optical coherence tomography
NPs: Nanoparticles
PAT: Photoacoustic tomography
2D: Two-dimensional
3D: Three-dimensional
S/B: Signal-to-background ratio
CCD: Charge-coupled device
CMOS: Complementary metal oxide sensor
SWIR: Short-wave infrared
QDs: Quantum dots
IVIS: In Vivo Imaging System
QY: Quantum yield
Cy7: Cyanine derivative dye
Tar-Cy7: Cy7-conjugated, targeted single-chain variable fragment antibody
Mut-Cy7: Cy7-conjugated, mutated single-chain variable fragment antibody
Cy7-CCPM: Cy7-labelled core-crosslinked polymeric micelles
BODIPYs: Boron-dipyrromethene
MB: Methylene blue
D–A–D: Donor–acceptor–donor
EGFR: Epidermal growth factor receptor
EDC: N-Ethyl-N′-(3-dimethylaminopropyl) carbodiimide hydrochloride
VEGF-A: Vascular endothelial growth factor-A
PEG: Polyethylene glycol
BSA: Bovine serum albumin
NC: Nanocapsules
CD: Carbon dots
SWNTs: Single-wall carbon nanotubes
cRGD: Cyclic pentapeptide
NIR-FME: NIR fluorescence molecular endoscopy
FLARE: Fluorescence-assisted resection and exploration
NIRAF: Near-infrared autofluorescence
trNIRS: Time-resolved near-infrared spectroscopy
PMX: Polymyxins
